# Biomass recovery of coastal young mangrove plantations in Central Thailand

**DOI:** 10.1038/s41598-024-61979-3

**Published:** 2024-05-18

**Authors:** Toshiyuki Ohtsuka, Suthathip Umnouysin, Vilanee Suchewaboripont, Nada Yimatsa, Chadtip Rodtassana, Morimaru Kida, Yasuo Iimura, Shinpei Yoshitake, Nobuhide Fujitake, Sasitorn Poungparn

**Affiliations:** 1 River Basin Research Center, Gifu University, 1-1 Yanagito, Gifu City, Gifu 501-1193 Japan; 2 Department of Biology, Faculty of Science, Silpakorn University, Nakhon Pathom, 73000 Thailand; 3 The Institute for the Promotion of Teaching Science and Technology, Bangkok, 10110 Thailand; 4 Department of Botany, Faculty of Science, Chulalongkorn University, Bangkok, 10330 Thailand; 5 Soil Science Laboratory, Graduate School of Agricultural Science, Kobe University, 1-1 Rokkodai, Nada, Kobe, Hyogo 657-8501 Japan; 6 School of Environmental Science, The University of Shiga Prefecture, 2500 Hassaka, Hikone, Shiga 522-8533 Japan; 7 Faculty of Education and Integrated Arts and Sciences, Waseda University, 2-2 Wakamatsu, Shinjuku, Tokyo 162-0056 Japan

**Keywords:** Aboveground biomass, *Avicennia alba*, Coarse root, Coastal degraded area, Fine root biomass, Forest ecology, Forest ecology

## Abstract

Around one-third of the world’s most carbon-rich ecosystems, mangrove forests, have already been destroyed in Thailand owing to coastal development and aquaculture. Improving these degraded areas through mangrove plantations can restore various coastal ecosystem services, including CO_2_ absorption and protection against wave action. This study examines the biomass of three coastal mangrove plantations (*Avicennia alba*) of different ages in Samut Prakarn province, Central Thailand. Our aim was to understand the forest biomass recovery during the early stages of development, particularly fine root biomass expansion. In the chronosequence of the mangrove plantations, woody biomass increased by 40% over four years from 79.7 ± 11.2 Mg C ha^-1^ to 111.7 ± 12.3 Mg C ha^−1^. Fine root biomass up to a depth of 100 cm was 4.47 ± 0.33 Mg C ha^−1^, 4.24 ± 0.63 Mg C ha^−1^, and 6.92 ± 0.32 Mg C ha^−1^ at 10, 12, and 14 year-old sites, respectively. Remarkably, the fine root biomass of 14-year-old site was significantly higher than those of the younger sites due to increase of the biomass at 15–30 cm and 30–50 cm depths. Our findings reveal that the biomass recovery in developing mangrove plantations exhibit rapid expansion of fine roots in deeper soil layers.

## Introduction

Tropical and subtropical mangrove forests are renowned as the most carbon (C)-rich ecosystems globally, with average ecosystem C storage being 2.5–5.0-fold higher than that of typical upland forests^1^. This is primarily due to the substantial soil organic carbon (SOC) pool in mangrove forests, with SOC in the top meter of soil accounting for 77% of the ecosystem C stocks^2^. However, mangrove forests face significant threats causing 30%–50% of loss of their coverage in the past 50 years due to coastal development and aquaculture^3^. In Southeast Asia, the loss of mangrove forests is ongoing, with an annual decline rate of 3.6%–8.1% in the twenty-first century^4^. Therefore, the conservation and restoration of mangrove forests not only serve to restore coastal ecosystem services, such as wave protection and food supply, but also provide a cost-effective means of mitigating climate change in Southeast Asian countries^5^. For example, although mangroves constitute only approximately 2.6% of total forest area in Indonesia, their degradation and deforestation contribute to approximately 10% of greenhouse gas emissions originating from the forestry sector^6^.

In Thailand, one-third of its mangrove forests have already been destroyed^7^ mainly due to shrimp farming^5^. Additionally, as the productivity of old aquacultural sites continues to decline, they are increasingly being abandoned throughout the country^8^. Consequently, there is a growing effort to promote the restoration of secondary mangrove forests and mangrove plantations in these degraded coastal areas. However, research on the process of restoring the C pool in mangrove ecosystems in abandoned areas is still in its early stages, in contrast to studies on C losses from mangrove deforestation (e.g.,^9–11^ ). Elwin et al.^8^ compared the ecosystem C stocks of abandoned shrimp ponds of different ages in Thailand and found a positive recovery trajectory for surface soil C through the natural regeneration of mangrove forests without active restoration efforts. Similarly, Osland et al.^12^ investigated ecosystem development following tidal wetland creation in Tampa Bay, Florida. Over a 20-year chronosequence, these authors observed a vegetation transition from salt marsh to naturally recruited mangrove forests and noted that soil organic matter increased with age alongside mangrove forest growth.

Although these pioneer studies examined regenerated mangrove forests using comparative approaches across sites of varying stand ages, they were conducted in scattered remote locations where environmental factors, such as soil properties and hydraulic conditions, as well as the initial degree of mangrove destruction, may differ. Lal^13^ cautioned that the “space-for-time substitution” (chronosequence) approach can lead to erroneous C stock estimations if soil characteristics vary among sites. Consequently, in recent years, there has been an increase in chronosequence studies focusing on adjacent mangrove plantations of different ages (e.g.,^14–16^), which provide insights into biomass recovery during the early stages of development. Additionally, although several studies have investigated the recovery of ecosystem C storage, including SOC, in both mangrove plantations^17^ and naturally regenerated mangroves^18,19^.

Compared to upland forests, the fine roots in mangrove forests are known to be distributed at greater soil depths^20–22^. For example, Kida et al.^23^ conducted soil sampling to a depth of 3.5 m to estimate SOC stocks in secondary mangrove forests in Thailand; fine root detritus was found even at depths greater than 2 m. Thus, although their role as C stock is small, fine roots contribute significantly to SOC accumulation in mangroves due to high production and low decomposition rates^22,24,25^. However, few studies have investigated the increase in fine root biomass in young mangroves, especially in deeper soil layers, despite root zone expansion being key to SOC accumulation.

The coastal area of the Bangpu Recreation Center in Samut Prakarn province located at the mouth of the Chao Phraya River, Central Thailand, was once covered by extensive mangrove forests that were devastated by industrial and urban development^26^. Annual mangrove plantations have been initiated since 2005 in various designated areas along the shoreline^27^. The adjacent mangrove plantations of different ages in Bangpu are an ideal target for a chronosequence approach to investigate the ecological restoration of C stocks in Southeast Asia. Therefore, the objective of this research was to determine the accumulation of woody biomass and the expansion of fine roots, in the early stages of coastal mangrove plantations using a chronosequence approach. We hypothesized that the recovery of ecosystem C storage in developing mangrove plantations is facilitated by the rapid growth of fine roots in deeper soil layers.

## Materials and methods

### Study site

**Figure 1:**
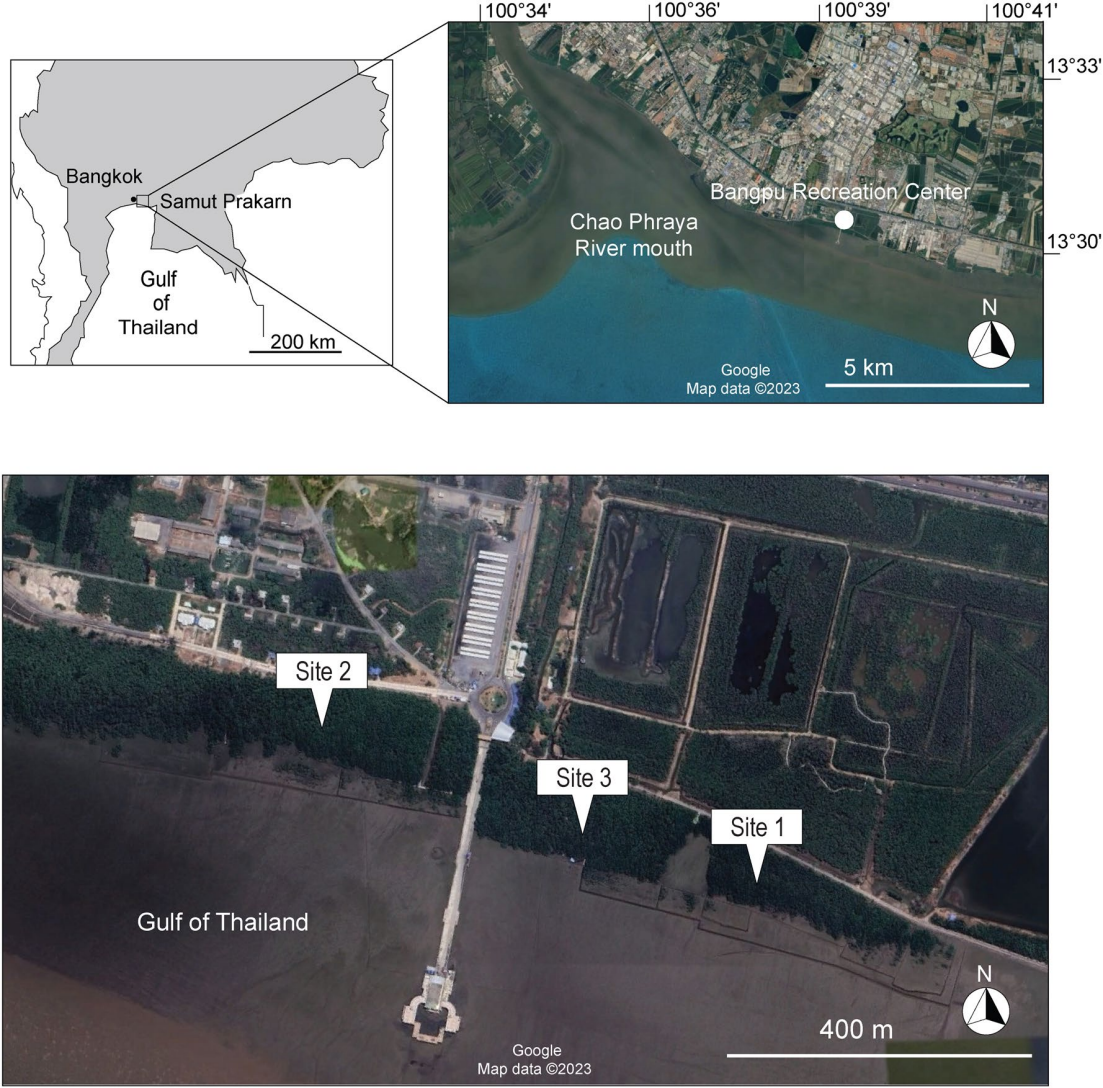
Locations of the study sites in Samut Prakan province, Central Thailand. Three parallel *Avicennia alba* mangrove plantations (Site 1–3) with different ages, located along the coastal line, were selected for this study. The source of satellite images is available at: http://www.google.com/earth/index.html (Accessed 29 June 2023).

The study was conducted in mangrove plantations located in Samut Prakarn province, Central Thailand (13°31′N, 100°39′E), specifically at the Bangpu Recreation Center on the eastern shore of the Chao Phraya River mouth (Fig. 1). The mangrove restoration project in the coastal fringe was initiated through collaboration between the Quartermaster Department of the Royal Thai Army in Samut Prakarn province and the Foundation for Environmental Education for Sustainable Development^27^. Mangrove plantations were started in 2005 to provide environmental education for local students, and subsequent plantings have taken place almost every year, resulting in plantations of different ages existing adjacent to each other along the coast (Fig. 1). Mixture of mangrove seedlings, *Avicennia alba, Rhizophora mucronata*, *R. apiculata* and *Sonneratia caseolaris* (aged 6–12 months), were systematically planted at 1 m intervals (approximately 10,000 stems ha^−1^) in abandoned coastal areas along the seashore. However, almost all stems except for *A. alba* already died out. The exact area and survival rate of each plantation has not been recorded. Notably, the plantations are occasionally used by local people for crab catching. Three parallel sites of plantations of different ages along the coastline (Fig. 1) were selected: Site 1 (planted in 2013), Site 2 (planted in 2011), and Site 3 (planted in 2009). The oldest site (planted in 2005) was excluded from this chronosequence approach because of the presence of remnant large mangrove trees in the plantation.

The study site experiences a tropical monsoon climate characterized by distinct rainy (May–October) and dry (November–April) seasons. The rainy season accounts for 82.5% of the annual rainfall, which amounts to 1008 mm. The mean annual temperature is 28.9 °C, with the highest and lowest temperatures occurring in May (30.5 °C) and January (26.8 °C), respectively^27^.

### Calculation of tree biomass

To estimate the mean forest biomass of each site, four randomly placed plots (10 × 10 m) were established. In May 2021, the diameter at breast height (DBH) was measured for all tree stems with a DBH ≥ 4.5 cm. Tree height (H) was also measured using a pole for all tree stems. To estimate the mean aboveground biomass (AGB) and coarse root biomass (CRB) for each site, the common allometric equations for mangrove species^28^ were used:

AGB = 0.251 *ρ* D^2.46^,

CRB = 0.199 *ρ*^0899^ D^2.22^,

where *ρ* represents the stem wood density (kg m^−3^) with bark, and D is the DBH (cm). The *ρ* value for *A. alba* is 0.506^28^.

We examined the relationship between the age of young mangrove plantations (aged < 30 years) similar to our study sites (the coastal fringe and oceanic sites) and their AGB (Mg C ha^−1^). The data are based on the studies of Cameron et al.^29^ (including secondary citations for review data) and recent findings^14,15,17,30,31^, as well as the present study. Tree carbon was calculated by multiplying biomass by a factor of 0.48^27^ for the dry weight–based biomass data, including our own data.

### Calculation of fine root biomass

We used the soil coring method to estimate fine root biomass in the three sites during February 2023, two years after the biomass measurements. Six soil samples were randomly collected in each site using a Handy Geoslicer (Fukken Co., Ltd., Hiroshima, Japan), which can extract a soil profile with a cross-sectional area of 27 cm^2^ (approximately 3 × 9 cm) and a depth of 100 cm while minimizing compaction. Each sample was divided into five soil layers (0–15, 15–30, 30–50, 50–75, and 75–100 cm) in situ.

All soil samples were transported to the laboratory and stored at a low temperature (5 °C). Subsequently, the roots from each sample were washed in a sieve (mesh size: 0.5 mm) using tap water and sorted manually into categories of living fine roots, living coarse roots, and dead roots based on their color and firmness. Coarse and fine roots were defined as roots with diameters of > 2 mm and ≤ 2 mm, respectively^32^. For samples collected from the 0–15 cm layer, aboveground roots (if present) were separated and removed prior to root washing. Prior to biomass measurements, all root samples were oven-dried at 60 °C until a constant weight was reached.

### Statistical analysis

One-way analysis of variance (ANOVA) was performed to test differences of forest structures and woody biomass among the sites with different ages. Differences in fine root biomass among the sites with different soil depths were also assessed using ANOVA. Subsequently, a post-hoc Tukey HSD test was used to identify significant differences among the sites. The significance threshold (*p*) for all tests was set at 0.05. All statistical analyses were carried out with the R programming language (Supplementary Information)^33^.

## Results

### Forest structure

**Figure 2:**
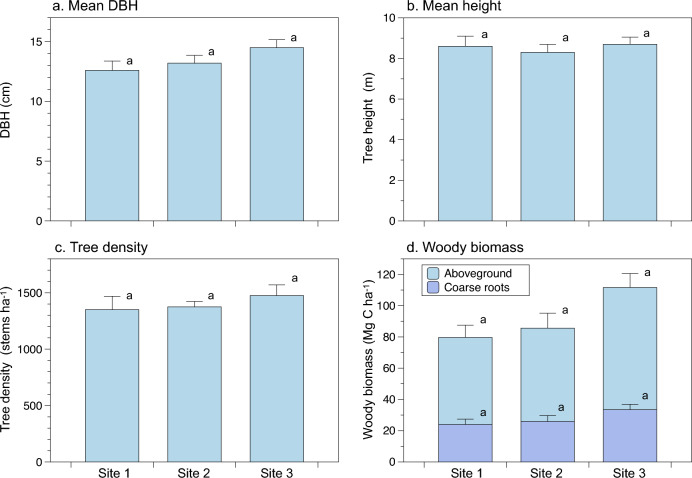
Change of forest structure (**a**-**c**) and woody biomass (**d**) in young *Avicennia alba* mangrove plantations (Site 1–3) along a chronosequence at Bangpu, Central Thailand (Mean ± SE). There were no significant differences across different sites using ANOVA.

According to the analysis of the chronosequence of forest structures, the mean DBH of planted mangroves tended to increase with the increasing stand age, although there were no statistical differences among the sites (*F*_*2,165*_ = 1.96, *p* = 0.14) (Fig. 2a). Canopy trees did not grow much with height during 8 to 12 years with a peak height of approximately 14 m (*F*_*2,164*_ = 0.199, *p* = 0.82) (Fig. 2b). Tree density already decreased in the 8-year-old plantations and had no significant change with age (*F*_*2,9*_ = 0.516, *p* = 0.61) (Fig. 2c).

### Woody biomass and fine root biomass

The woody biomass tended to increase with the increasing stand age; 79.7 ± 11.2 Mg C ha^−1^, 85.6 ± 13.3 Mg C ha^−1^, and 111.7 ± 12.3 Mg C ha^−1^ at Site 1, 2, and 3, respectively (Fig. 2d). While there were no statistical differences of total woody biomass among the sites (*F*_*2,9*_ = 1.92, *p* = 0.20). AGB were 55.7 ± 7.80 Mg C ha^−1^, 59.7 ± 9.55 Mg C ha^−1^, and 78.3 ± 8.84 Mg C ha^−1^ at Site 1, 2, and 3, respectively, with no statistical differences.

The fine root biomass in each site exhibited a clear decreasing pattern at depths of up to 100 cm (Table 1), although the fine roots were still present at depths greater than 75 cm in all sites. The total fine root biomass up to 100 cm showed no significant difference between Site 1 (4.47 ± 0.33 Mg C ha^−1^) and Site 2 (4.24 ± 0.63 Mg C ha^−1^) (Table 1), but the fine root biomass of Site 3 (6.92 ± 0.32 Mg C ha^−1^) was significantly higher than the others (*F*_*2,15*_ = 10.72, *p* = 0.0013). The fine root biomass at the depth of 15–30 cm (*F*_*2,15*_ = 14.45, *p* < 0.001) and 30–50 cm (*F*_*2,15*_ = 6.00, *p* = 0.012) significantly increased with stand age, although that in the surface soil (0–15 cm) was no difference among the sites (*F*_*2,15*_ = 1.65, *p* = 0.22) (Table 1).

**Table 1:** Fine root biomass (g m^−2^) at different depths in young *Avicennia alba* mangrove plantations at Bangpu in Central Thailand.

Soil depth (cm)	Site 1 (10 yrs)	Site 2 (12 yrs)	Site 3 (14 yrs)	*p* value
	Mean ± SE	Mean ± SE	Mean ± SE	
0–15	546.3 ± 65.6	618.4 ± 103.9	744.5 ± 56.2	0.22
15–30	298.8 ± 24.7^a^	183.4 ± 43.8^a^	521.9 ± 60.2^b^	< 0.001
30–50	110.0 ± 19.6^a^	105.2 ± 25.0^a^	217.1 ± 31.5^b^	0.012
50–75	58.4 ± 5.3	54.6 ± 10.6	89.1 ± 17.1	0.12
75–100	25.5 ± 3.7	24.4 ± 4.3	36.0 ± 5.4	0.17
0–100	1039.0 ± 77.7^a^	986.1 ± 147.5^a^	1608.7 ± 74.5^b^	0.0013
0–100 (Mg C ha^−1^)	4.47 ± 0.33	4.24 ± 0.63	6.92 ± 0.32	

Different letters indicate significant differences in each soil depth across different sites based on a post-hoc test. Fine root C mass (Mg C ha^−1^) was calculated by multiplying the biomass by a factor of 0.43^41^.

## Discussion

**Figure 3:**
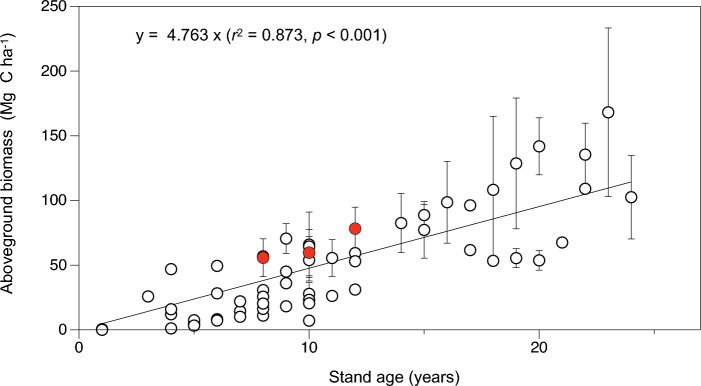
Relationship between the age of young mangrove plantations (years) along the coastal fringe and their aboveground biomass (Mg C ha^−1^ ± SD). The data are based on the studies of Cameron et al.^29^ (including secondary citations for review data), Cuc & Hien^15^, Phan et al.^30^, Uddin et al.^17^, Yu et al.^14^ and Zhang et al.^31^, as well as the present study (red circles). Tree carbon was calculated by multiplying biomass by a factor of 0.48 for the dry weight–based biomass data, including our own data.

Numerous studies have examined biomass recovery in mangrove plantations and restored natural secondary forests (e.g.^8,16,18^), where biomass significantly increases with increasing stand age during the development stages. However, the extent of biomass recovery varies depending on site conditions. For example, Cameron et al.^29^ compared C stocks in restored mangroves at two contrasting sites in Sulawesi, Indonesia, and revealed that a site with deep muds and silty substrates promote higher rates of biomass compared with a site with coastal fringing and oceanic sites. Therefore, we additionally examined the AGB in various stand ages of young plantations (aged < 30 years) at only coastal fringing and oceanic sites, based on data from Cameron et al.^29^ and recent findings^14,15,17,30,31^. The AGB (Mg C ha^−1^) of mangrove plantations significantly increased with age (*r*^*2*^ = 0.87, *p* < 0.001), and AGB values of our study sites were relatively high (Fig. 3). One possible explanation for the high biomass recovery at our study site is the influence of the planted species. Previous studies mainly focused on *Rhizophora apiculata* and/or *R. mucronata*, as these species produce long hypocotyl seedlings that are convenient for transportation, carrying, and planting. *Avicennia alba* is a pioneer species that dominates open areas along rivers and seaward edges^34,35^ and may have a higher growth rate than *Rhizophora* plantations. Additionally, the soil conditions of the muddy coastal fringe at the Bangpu site (with > 82% silt)^27^ may be more favorable compared with typical sandy coastal fringing sites. However, there were no significant differences in woody biomass in the chronosequence of the present study, despite an increasing trend (Fig. 2d). Therefore, measurements with more replicates, using rather large plots, should be carried out to reduce the error in the estimation of woody biomass.

Comparatively studying mangrove fine root biomass among sites is challenging compared to woody biomass owing to variations in depth and inclusion of necromass^22,36^. Adame et al.^37^ reviewed the fine root biomass in mangroves, revealing a wide variation of 0.61–91.4 Mg ha^−1^ using coring methods. They stated that studies integrate over depths shallower than 45 cm are likely underestimating total root biomass, because most root biomass is concentrated in the first meter of soil, with > 50% of total root biomass in the first 45 cm depth. In our study sites, the fine root biomasses were 8.0–12.7 Mg ha^−1^ and 9.9–16.1 up to depths of 30 cm and 100 cm, respectively (Table 1). 21.2% of fine roots were present at depths deeper than 30 cm in the 14-year-old plantation, and 18.7% even in the 10-year-old plantation.

Moreover, fine root necromass is significantly more abundant in mangrove soil than in upland forests^22^, leading to large errors in fine root biomass estimation. Two particularly high fine root biomass values in the reviewed data^37^ were 91.4 Mg ha^-1^ (up to 30 cm in Thailand) and 76.2 Mg ha^-1^ (up to 100 cm in New Zealand), with both datasets including live and dead fine roots. From the dataset in Thailand^38^, more than 95% of the fine roots were necromass, resulting in an actual fine root biomass of 1.37 ± 0.94 Mg ha^−1^. Separation of live and dead roots is crucial for estimating of fine root biomass, and standardized methods such as density-based method^39^ should be considered for comparisons. Our findings reveal that the fine root biomass increases significantly during the early stages of mangrove plantations in the depth of 15–30 cm and 30–50 cm soil layers. This suggests that the increase in fine root biomass at greater depths (15–50 cm) contributed significantly to the total fine root biomass with stand age.

In addition to forest biomass, SOC is known to recover rapidly in young mangrove forests^12^. For instance, SOC stocks in the surface layers of naturally regenerated mangroves in French Guiana increased from 4.8 Mg C ha^−1^ (3 years) to 10.36 Mg C ha^−1^ (9 years)^40^. The abundant distribution of live and dead fine roots resulting from high fine root production and turnover is crucial for SOC stocks in mangroves^22,24^. Kida et al.^23^ conducted a SOC composition analysis of a secondary mangrove forest up to a depth of 3.5 m and performed a principal component analysis of the resulting compositional data. Their results revealed that root abundance had a stronger influence than soil texture on the abundance and composition of mangrove SOC. The rapid expansion of fine roots in a deeper direction would contribute to SOC stocks in young mangrove plantations, and thus, not only the measurements of fine root abundance but also investigation of necromass and fine root decomposition is necessary to understand the process.

## Conclusion

The development of biomass increment in young mangrove plantations in central Thailand provides valuable insights into the changes in biomass C stocks during mangrove restoration. Our findings demonstrate that both woody biomass and fine root biomass exhibit rapid increases with stand age of mangrove plantations along the coastal fringe. Additionally, the fine root growth had reached up to a depth of 1 m even in 10-year-old plantations, and significantly increased not in the surface soil but in the depth of 15 cm to 50 cm. Our results highlight the expansion of fine roots in a deeper direction in young mangrove plantations, emphasizing the importance of studying fine root dynamics in deeper soil layers to understand the mechanisms of ecosystem C recovery in mangrove ecosystems.

### Supplementary Information

Supplementary Information.

## Data Availability

The datasets of the current study are available in the supplementary information.
