# Supplementary material for: Biomass recovery of coastal young mangrove plantations in Central Thailand
Source: Sci Rep. 2024 May 18;14:11359. doi: 10.1038/s41598-024-61979-3 (PMC11102487; doi:10.1038/s41598-024-61979-3)
Supplement: Supplementary file 1 — Supplementary Information. [file 41598_2024_61979_MOESM1_ESM.zip › SUPPLEMENTARY INFORMATION.docx]

**SUPPLEMENTARY INFORMATION**

Biomass recovery of coastal young mangrove plantations in Central Thailand.

Toshiyuki Ohtsuka, Suthathip Umnouysin, Vilanee Suchewaboripont, Nada Yimatsa, Chadtip Rodtassana, Morimaru Kida, Yasuo Iimura, Shinpei Yoshitake, Nobuhide Fujitake, Sasitorn Poungparn

# Supplementary R code for Ohtsuka et al. (2024) Biomass recovery of coastal young mangrove plantations in Central Thailand. Scientific Reports

# Set your working directory

setwd("")

# Load the data set

forest <- read.csv("DBH&H.csv", header = TRUE)

density <- read.csv("Density.csv", header = TRUE)

biomass <- read.csv("Biomass.csv", header = TRUE)

root <- read.csv("Fine roots.csv", header = TRUE)

# ANOVA of forest structure among plots

AnovaModel.1 <- aov(DBH ~ Plot, data=forest)

summary(AnovaModel.1) # not significant

AnovaModel.2 <- aov(H ~ Plot, data=forest)

summary(AnovaModel.2) # not significant

# ANOVA of tree density among plots

AnovaModel.1 <- aov(Density ~ Plot, data=density)

summary(AnovaModel.1) # not significant

# ANOVA of plant biomass among plots

AnovaModel.1 <- aov(AGB ~ Plot, data=biomass)

summary(AnovaModel.1) # not significant

AnovaModel.2 <- aov(BGB ~ Plot, data=biomass)

summary(AnovaModel.2) # not significant

AnovaModel.3 <- aov(total ~ Plot, data=biomass)

summary(AnovaModel.3) # not significant

# ANOVA of plant biomass among plots

AnovaModel.1 <- aov(Total ~ Plot, data=root)

summary(AnovaModel.1) # significant

TukeyHSD(AnovaModel.1) # Site 3

AnovaModel.2 <- aov(X0.15.cm ~ Plot, data=root)

summary(AnovaModel.2) # not significant

AnovaModel.3 <- aov(X15.30.cm ~ Plot, data=root)

summary(AnovaModel.3) # significant

TukeyHSD(AnovaModel.3) # Site 3

AnovaModel.4 <- aov(X30.50.cm ~ Plot, data=root)

summary(AnovaModel.4) # significant

TukeyHSD(AnovaModel.4) # Site 3

AnovaModel.5 <- aov(X50.75.cm ~ Plot, data=root)

summary(AnovaModel.5) # not significant

AnovaModel.6 <- aov(X75.100.cm ~ Plot, data=root)

summary(AnovaModel.6) # not significant
